# Importance of *c*-Type cytochromes for U(VI) reduction by *Geobacter sulfurreducens*

**DOI:** 10.1186/1471-2180-7-16

**Published:** 2007-03-08

**Authors:** Evgenya S Shelobolina, Maddalena V Coppi, Anton A Korenevsky, Laurie N DiDonato, Sara A Sullivan, Hiromi Konishi, Huifang Xu, Ching Leang, Jessica E Butler, Byoung-Chan Kim, Derek R Lovley

**Affiliations:** 1 Department of Microbiology, University of Massachusetts, Amherst, MA, USA; 2 Department of Microbiology, College of Biological Science, University of Guelph, Guelph, Ontario, Canada; 3 Department of Geology and Geophysics, University of Wisconsin, Madison, WI, USA

## Abstract

**Background:**

In order to study the mechanism of U(VI) reduction, the effect of deleting *c*-type cytochrome genes on the capacity of *Geobacter sulfurreducens *to reduce U(VI) with acetate serving as the electron donor was investigated.

**Results:**

The ability of several *c*-type cytochrome deficient mutants to reduce U(VI) was lower than that of the wild type strain. Elimination of two confirmed outer membrane cytochromes and two putative outer membrane cytochromes significantly decreased (ca. 50–60%) the ability of *G. sulfurreducens *to reduce U(VI). Involvement in U(VI) reduction did not appear to be a general property of outer membrane cytochromes, as elimination of two other confirmed outer membrane cytochromes, OmcB and OmcC, had very little impact on U(VI) reduction. Among the periplasmic cytochromes, only MacA, proposed to transfer electrons from the inner membrane to the periplasm, appeared to play a significant role in U(VI) reduction. A subpopulation of both wild type and U(VI) reduction-impaired cells, 24–30%, accumulated amorphous uranium in the periplasm. Comparison of uranium-accumulating cells demonstrated a similar amount of periplasmic uranium accumulation in U(VI) reduction-impaired and wild type *G. sulfurreducens*. Assessment of the ability of the various suspensions to reduce Fe(III) revealed no correlation between the impact of cytochrome deletion on U(VI) reduction and reduction of Fe(III) hydroxide and chelated Fe(III).

**Conclusion:**

This study indicates that *c*-type cytochromes are involved in U(VI) reduction by *Geobacter sulfurreducens*. The data provide new evidence for extracellular uranium reduction by *G. sulfurreducens *but do not rule out the possibility of periplasmic uranium reduction. Occurrence of U(VI) reduction at the cell surface is supported by the significant impact of elimination of outer membrane cytochromes on U(VI) reduction and the lack of correlation between periplasmic uranium accumulation and the capacity for uranium reduction. Periplasmic uranium accumulation may reflect the ability of uranium to penetrate the outer membrane rather than the occurrence of enzymatic U(VI) reduction. Elimination of cytochromes rarely had a similar impact on both Fe(III) and U(VI) reduction, suggesting that there are differences in the routes of electron transfer to U(VI) and Fe(III). Further studies are required to clarify the pathways leading to U(VI) reduction in *G. sulfurreducens*.

## Background

Uranium is a long-lived radionuclide that poses an ecological and human health hazard. The use of uranium in nuclear fuels and nuclear weapons production has created a large amount of nuclear waste, and the disposal of nuclear waste in near-surface environments remains a serious environmental issue. In particular, uranium from radioactive waste deposits can leak into the groundwater system. In order to prevent further contamination of aquifers with uranium and halt the expansion of uranium contaminated ground water plumes, it is necessary to immobilize uranium in a geochemically inert form *in situ *[1-4]. Stimulation of the microbial reduction of soluble hexavalent uranium U(VI) to tetravalent uranium U(IV) which precipitates as the mineral uraninite, has been proposed as a method for the immobilization of uranium *in situ *[5]. Stimulation of dissimilatory metal reduction in laboratory incubations of uranium contaminated sediment [6] and in a uranium contaminated aquifer during *in situ *uranium bioremediation field trials [7-9] resulted in the concomitant removal of soluble, hexavalent U(VI) from the ground water and domination of the microbial community by indigenous Fe(III)-reducing bacteria belonging to the family *Geobacteraceae *of the delta subdivision of the *Proteobacteria*.

Little is known about the mechanism of microbial U(VI) reduction [10], however, *c*-type cytochromes are thought to play a key role in this process. Biochemical [11] and genetic [12,13] studies performed on *Desulfovibrio *species, have suggested that a periplasmic *c*3 cytochrome is required for U(VI) reduction. The ability of U(VI) to oxidize *c*-type cytochromes in intact *Geobacter metallireducens *cells provided circumstantial evidence for the involvement of *c*-type cytochromes in electron transfer to U(VI) [14] in *Geobacter *species as well. A role for cytochromes in U(VI) reduction was further supported by the finding that deletion of the gene encoding the periplasmic *c*_7 _cytochrome, PpcA, negatively impacted acetate-dependent U(VI) reduction in *G. sulfurreducens*. Finally, a recent study on the mechanism of U(VI) reduction by *S. oneidensis *strain MR-1 confirmed that *c*-type cytochromes are essential for U(VI) reduction by this species. [15].

The subcellular localization of microbial U(VI) reduction is also unclear. The detection of copious amounts of extracellular uraninite precipitate during early studies of U(VI) reduction in *G. metallireducens *[16] suggested that U(VI) reduction might take place at the cell surface, the likely site of Fe(III) reduction [17]. *Desulfovibrio desulfuricans *also produces extracellular uraninite [18]. However, accumulation of extracellular uraninite does not preclude a periplasmic location for U(VI) reduction, because the initial products of enzymatic U(VI) reduction are small (from 1–5 to 200 nm) [11,15,16,19,20] and could conceivably diffuse out of the periplasm prior to forming larger extracellular precipitates. In fact, subsequent studies performed on *G. sulfurreducens *provided evidence for periplasmic U(VI) reduction including detection of U(IV) precipitate within the periplasm of thin-sections of cells actively reducing U(VI) and failure of proteolytic treatment of intact cells to inhibit U(VI) reduction while inhibiting Fe(III) oxide reduction [21]. In a recent study on the role of cytochromes in U(VI) reduction by *Shewanella oneidensis *strain MR-1, UO_2 _nanoparticles were detected both in the periplasm and outside of resting cell suspensions exposed to 10 mM lactate and 250 μM U(VI) for 24 hours [15]. Genetic studies indicate that both periplasmic and outer membrane cytochromes may play a role in U(VI) reduction. In *Desulfovibrio *species and in *G. sulfurreducens *knocking out a periplasmic *c*-type cytochrome significantly impaired uranium reduction [12,21]. In *S. oneidensis *strain MR-1, knocking out two outer membrane cytochromes, MtrC and OmcA significantly reduced U(VI) reduction [15].

Multiple *c*-type cytochrome-deficient strains of *G. sulfurreducens*, a genetically tractable *Geobacter *species with a sequenced genome [22,23] were constructed as part of a genetic investigation of the role of *c*-type cytochromes in Fe(III) reduction [24-28]. These strains had varying degrees of impairment in Fe(III) reduction [24-28] and included strains deficient in both outer membrane [24,25] and periplasmic cytochromes [26-29]. This strain collection, therefore, constituted a uniquely suitable system for investigating three aspects of U(VI) reduction: 1) the role of *c*-type cytochromes in U(VI) reduction, 2) the subcellular localization of U(VI) reduction, and 3) the relationship between the electron transport pathways to Fe(III) and U(VI). Here we report on the assessment of the Fe(III) and U(VI) capabilities of twelve cytochrome-deficient strains as well as the detection of periplasmic uranium accumulation in strains impaired in U(VI) reduction.

## Results and Discussion

### Development of a U(VI) reduction assay for *G. sulfurreducens*

**Figure 1:**
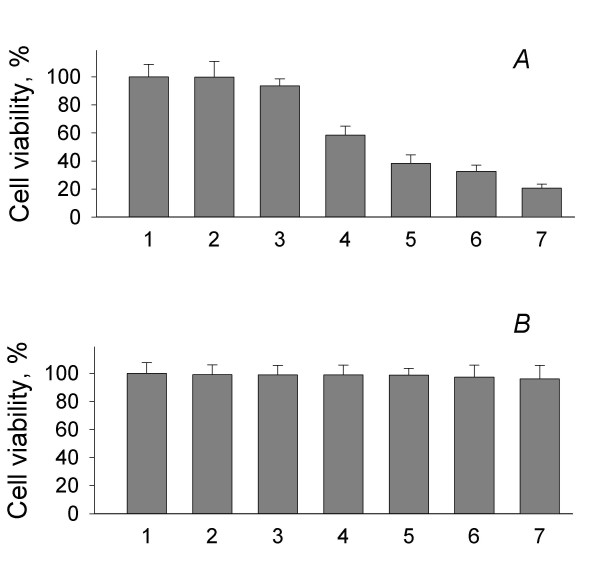
**Cell viability in previously published (A) and modified (B) U(VI) reduction assays**: **1 **– culture; **2 **– washed cells suspended in washing buffer; **3 **– cells suspended in reaction buffer (t = 0 hours); **4 **– t = 1 hour; **5 **– t = 2 hours; **6 **– t = 3 hours; **7 **– t = 4 hours. Data are means of duplicates ± SD

The preservation of cell viability is crucial for assessing the capacity for U(VI) reduction, because lysed cells release intracellular components many of which have the capacity to nonspecifically reduce U(VI) [10]. When the viability of cell suspensions prepared using the previously published protocol was tested, it was found that the majority of the cells, 79.3 ± 2.9% (Fig. 1), were no longer viable at the end of a 4 hr U(VI) reduction assay. As the result of modifications to this protocol, loss of viability was essentially eliminated; 96.1 ± 9.1% of the cells remained viable over the course of the 4 hr incubation (Fig. 1). Viability was preserved by minimizing the difference in osmotic pressure between the growth medium and the buffers used to wash and incubate cell suspensions and by decreasing the amount of biomass added to incubations.

**Figure 2:**
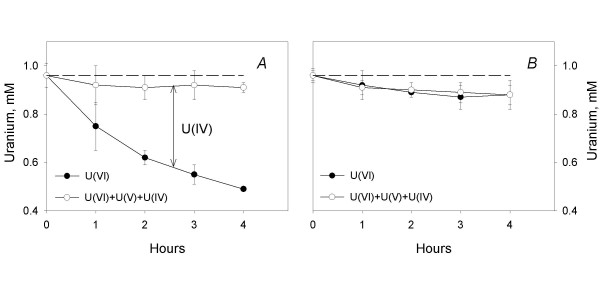
**Measurement of U(VI)-reducing activity by resting (A) and killed (B) cells of the strain DL1**. Final protein concentration is 0.014 mg/ml; data are means of triplicates ± SD.

Enzymatic uranium reduction was determined by subtracting U(VI) from the total amount of extractable uranium at each time point. Total uranium was quantitated by diluting samples withdrawn from the cell suspension in 100 mM bicarbonate and bubbling the samples for 15 min with air to convert any reduced uranium, U(IV) and U(V) [30], to U(VI) such that it could be detected by kinetic phosphorescence analysis (KPA). Surprisingly, the total amount of extractable uranium was 5–10% lower than the amount of U(VI) added to the cell suspension buffer at every point except for time zero (Fig. 2A). Further bubbling with air did not increase recovery of uranium (data not shown). The decline in the amount of total extractable uranium was similar in tubes that contained killed cells (Fig. 2B) and did not occur in reaction buffer incubated in the absence of cells (data not shown). This result indicated that decline in the total amount of extractable uranium was not due to an enzymatic mechanism and suggested that it was not biologically significant.

**Figure 3:**
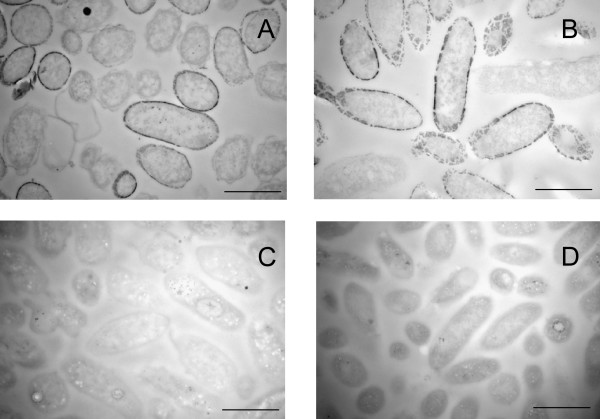
**Transmission Electron Microscopy of thin sections of the U(VI) reducing strains DL1 (A and C) and MacA (B and D) before (A, B) and after (C, D) treatment with bicarbonate and air**. Bar represents 1 μm

In order to evaluate the efficiency of the extraction procedure and gain insight into the nature of "missing" uranium, thin sections were prepared from cell suspensions of the wild type and MacA-deficent strains following exposure to 1 mM U(VI) and 5 mM acetate for 2 hours with and without bicarbonate extraction and bubbling with air. Both the wild type and the MacA-deficient strains accumulated uranium in the periplasm (Fig. 3A and 3B). Periplasmic uranium was not detected following treatment with bicarbonate and air (Fig. 3C and 3D). Therefore, the "missing" uranium did not appear to be intracellular.

Because the decline in the total amount of extractable uranium was not due to an enzymatic mechanism and did not appear to be due to failure to extract intracellular uranium, the rate of enzymatic U(VI) reduction was calculated by subtracting extractable U(VI) from the total amount of extractable uranium, U(VI) + U(V) + U(IV), rather than from the amount of U(VI) added to incubation.

The valence of the product of U(VI) reduction generated by *G. sulfurreducens *was not determined as part of this study. Although it is generally assumed that U(VI) is reduced to U(IV) via a two electron transfer, the product of U(VI) reduction by *G. sulfurreducens *could be U(IV) and/or U(V). EXAFS spectroscopy analysis has recently demonstrated that the initial product of U(VI) reduction by *G. sulfurreducens *was U(V), suggesting a one electron transfer [30]. The pentavalent UO_2 _^+ ^ion is unstable and can disproportionate into U(IV) and U(VI), however, as Renshaw et al [30] demonstrated, U(V) is still the main product after 4 hours of microbial reduction.

### Overview of cytochrome knockout strains

A collection of twelve cytochrome-deficient strains, consisting of eleven single mutants and one double mutant, in which two cytochrome genes were deleted simultaneously (Table 1), was analyzed in this study. These included four strains deficient in members of a family of five low molecular weight *c*_7_-type cytochromes (PpcA-E, Table 2), which have been proposed to function as periplasmic electron shuttles [27]. Although the periplasmic localization of only one member of this family, PpcA [31] has been confirmed, it is highly likely that PpcB, PpcC, and PpcD are also periplasmic, given the high degree of similarity between their predicted signal sequences and that of PpcA (75–100%). The signal sequence of PpcE is only 45% similar to that of PpcA. However, analysis of PpcE with the Proteome Analyst Specialized Subcellular Localization Server v2.5 [32,33] suggests that it too is likely to be periplasmic. Phenotypic analysis of the mutants deficient in the various Ppc cytochromes revealed a range of changes in the ability of *G. sulfurreducens *to reduce the soluble (chelated) form of iron, Fe(III)citrate, possibly due to compensatory interactions between members of this family of closely related cytochromes [28,34]. In contrast, deletion of an unrelated putative periplasmic cytochrome, MacA, nearly eliminated Fe(III) citrate reduction by *G. sulfurreducens*. MacA has been proposed to transfer electrons from the inner membrane to the periplasm [26], and expression of this loosely membrane associated cytochrome is induced during growth on Fe(III) citrate.

The Fe(III)-reducing phenotypes of four strains lacking outer membrane cytochromes (Table 2) have also been determined. These cytochromes include OmcE, which is loosely associated with the cell surface [35]; OmcB, which is exposed at the cell surface but is tightly associated with the outer membrane [36]; OmcC which has a signal sequence that is 100% identical to that of OmcB; and OmcF, an outer membrane associated cytochrome for which the orientation remains to be determined [24]. OmcE was reported to be required for the reduction of insoluble Fe(III) hydroxide but not soluble Fe(III) [35], whereas OmcB and OmcF, were required for the reduction of both forms of Fe(III) [24,25]. In contrast, deletion of OmcC did not significantly impair the reduction of either form of iron [25].

This work represents the first genetic characterization of one putative periplasmic cytochrome, GSU0616, and two putative outer membrane cytochromes, GSU0332 and GSU1334.

### Impact of cytochrome knockouts on U(VI) reduction

**Figure 4:**
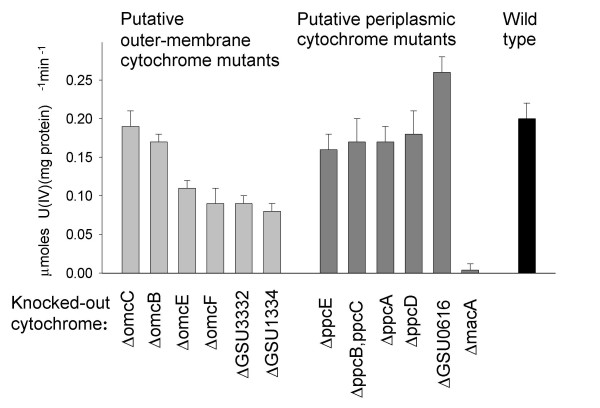
**U(VI)-reducing activity of wild type and mutant strains of *G. sulfurreducens***. Data are means of triplicates ± SD.

On average, the impact of knocking out confirmed or putative outer membrane cytochromes on the rate of U(VI) reduction was greater than that of eliminating periplasmic cytochromes (Fig. 4). However, there was a wide range of U(VI)-reducing phenotypes within both groups. Among the periplasmic cytochromes, only MacA appeared to play a significant role in U(VI) reduction. Deletion of *macA*, which is also essential for Fe(III) reduction [26], decreased the rate of U(VI) reduction by 98%. In contrast, the U(VI)-reducing activity of the strain deficient in putative periplasmic cytochrome, GSU0616, was actually 30% greater than that of wild type.

The U(VI)-reducing activities of the various members of the Ppc family of cytochromes, which are structurally related to the *c*_3 _cytochromes that have been implicated in U(VI) reduction in *Desulfovibrio *species [37,38], were 80–90% of wild type. These results contrast with those of a previous report [27], in which deletion of the *ppcA *gene was shown to decrease acetate-dependent U(VI) reduction by 80% [27]. This difference may be due to modifications made to the cell suspension assay protocol, which greatly enhanced preservation of viability, or, alternatively, to differences in the expression of other genes, including the remaining Ppc family members, as a result of alterations in culturing conditions prior to the preparation of cell suspensions.

It was previously proposed that U(VI) reduction takes place in the periplasm, based on the detection of uraninite in the periplasm of U(VI)-reducing cells and the reported dramatic effect of *ppcA *deletion on U(VI) reduction [27]. However, in this report, elimination of two confirmed outer membrane cytochromes and two putative outer membrane cytochromes significantly decreased (ca. 50–60%) the ability of *G. sulfurreducens *to reduce U(VI) (Fig. 4). The ability to transfer electrons to U(VI) did not appear to be a general property of outer membrane cytochromes, as elimination of OmcB and OmcC had very little impact on U(VI) reduction. These results suggest that U(VI) reduction may, in fact, take place at the cell surface and may involve specific outer membrane cytochromes. Similar results were obtained in a recent study of U(VI)reduction by *S. oneidensis*, where two outer membrane cytochromes, MtrC and OmcA, were documented to be important for U(VI) reduction [15].

The lack of U(VI)-reducing activity in the MacA deficient mutant does not preclude the possibility of U(VI) reduction at the cell surface. Deletion of a periplasmic electron shuttle would be expected to impact both periplasmic and cell surface electron-transfer processes. In contrast, loss of an outer-membrane cytochrome would not be expected to impact periplasmic U(VI) reduction. The finding that knocking out OmcE, an outer membrane cytochrome reported to be loosely associated with the cell surface, decreased U(VI) reduction by *G. sulfurreducens *by 45% is a strong indication that U(VI) reduction may indeed occur at the cell surface.

### Uranium accumulation in the periplasm of *G. sulfurreducens*

In order to learn more about the relationship between uranium accumulation in the periplasm and the U(VI)-reducing activity of *G. sulfurreducens*, thin sections were prepared from wild type, MacA-deficient, and GSU1334-deficient suspensions that had been exposed to 1 mM U(VI) and 5 mM acetate for 2 hours and examined via transmission electron microscopy (TEM). Representative TEM images show cell wall outlines (with dark contrast) due to enrichment of uranium (Fig. 3).

Surprisingly, only 24% of wild type cells accumulated uranium in the periplasm (Fig. 3A). A similar percentage (30%) was observed for both mutant strains (Fig. 3B). The TEM observation that uranium accumulation was not uniform could have several possible causes. One possible explanation for variations in uranium accumulation among individual cells, is that only those cells that are actively reducing U(VI) accumulate periplasmic uranium. If this were the case, the number of cells accumulating uranium in the periplasm would correlate with the U(VI)-reducing activity of the various cell suspensions. There was, however, no significant difference in the relative abundance of cells accumulating periplasmic uranium in the wild type and mutant cultures (24% vs 30%) in our experiments.

Variations in uranium accumulation may reflect variations in the physiological state of individual cells. For example, Bayer and Bayer (1991) observed that when growing *Escherichia coli *cultures were treated with rare earth element ions, accumulation of lanthanides in the periplasm was associated with loss of cell viability [39]. In our experiments, the viability of the wild type cells was determined by acridine orange staining and averaged 98.8%, whereas the percentage of cells accumulating periplasmic uranium was 24%. Therefore, loss of viability does not explain the heterogenous periplasmic uranium accumulation detected by TEM. The acridine orange staining technique is dependent upon the integrity of the inner membrane [40], while periplasmic uranium accumulation is likely to be more dependent upon the integrity of the outer membrane. Thus cell to cell variations in periplasmic uranium accumulation may be due to differences in outer membrane permeability or to changes in membrane integrity that occur during preparation procedures prior to thin sectioning for electron microscopy.

**Figure 5:**
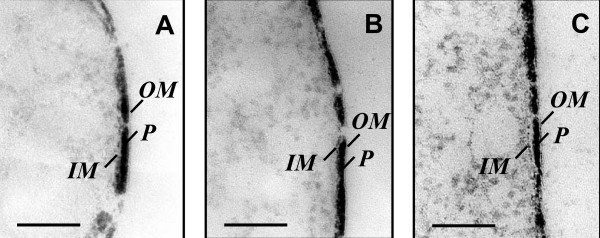
**Transmission Electron Microscopy of thin sections of U(VI) reducing *G. sulfurreducens***. **A**, **B **and **C**. Periplasmic uranium accumulation by various strains of *G. sulfurreducens*: **A **– wild type (DL1), **B **– DLMC5 (U(VI) reduction is inhibited 60%), **C **– DL1-MacA (U(VI)-reduction is inhibited completely). ***OM ***– outer membrane, ***P ***– periplasm, ***IM ***– inner membrane. Bar represents 0.1 μm.

When cells that accumulated uranium from the three strains were compared (Figures 5A–C), there were no apparent differences in the amount of uranium that accumulated in the periplasm. The MacA-deficient mutant, which is essentially incapable of U(VI) reduction, and the GSU1334-deficient mutant, which reduces uranium at only 50% of the wild type rate, accumulated as much uranium in the periplasm as wild type.

**Figure 6:**
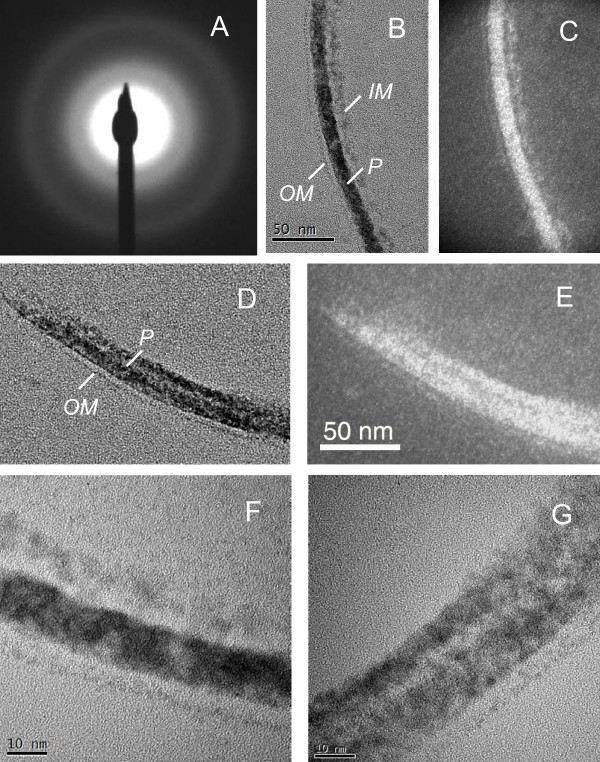
**High-resolution TEM (HRTEM) and selected-area electron diffraction (SAED) characterization of uranium-loaded periplasm of the DL1 and DL1-MacA strains**. **A**. representative SAED pattern from the cell wall displaying diffuse diffraction rings. Average scattering distance measured from the first diffraction ring is 2.2 Å; **B **and **C **– High magnification bright-field (BF) and dark-field (DF) TEM images of the cell wall of the strain DL1-MacA; **D **and **E **– BF and DF TEM images of the cell wall of the strain DL1; **F **and **G **– High-resolution TEM image of the cell wall of the strains DL1-Mac-1(**F**) and DL1 (**G**), showing amorphous features of the U-bearing cell wall layers. ***OM ***– outer membrane, ***P ***– periplasm, ***IM ***– inner membrane.

**Figure 7:**
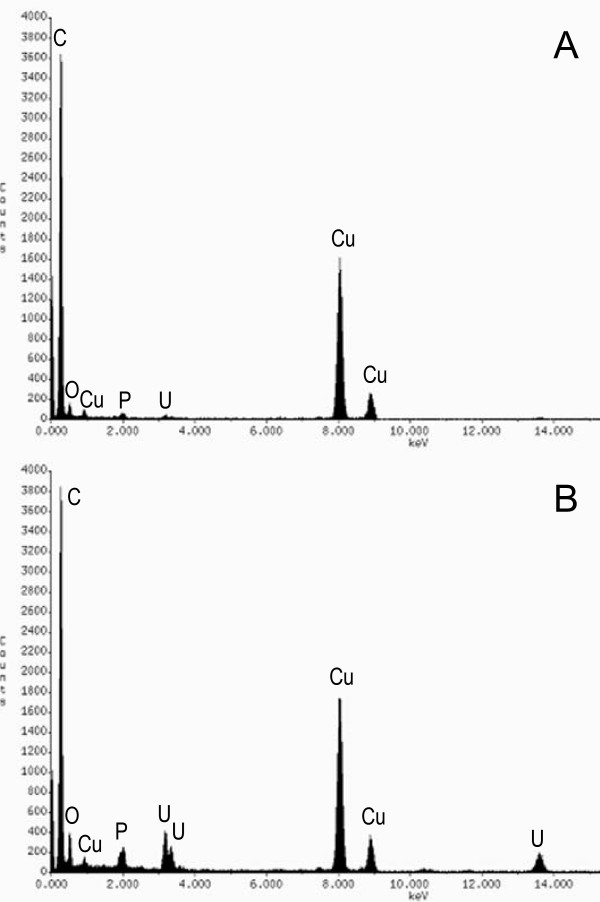
X-ray EDS spectra from the cytoplasm (A) and periplasm (B) of a U(VI)-reducing cell of the strain DL1.

The uranium present in the periplasms of the wild type (DL1) and MacA-deficient (DL1-MacA) cells was additionally characterized using high-resolution TEM (HRTEM), selected-area electron diffraction (SAED), and X-ray energy-dispersive spectroscopy (EDS), none of which showed significant differences between the form and distribution of the uranium present in the periplasm of the two strains. SAED patterns from the periplasmic space of both strains displayed two very diffuse rings, which indicated that the uranium-bearing layers were either non-crystalline or amorphous-like materials. A representative SAED pattern is shown in Fig. 6A. High magnification bright-field images of the DL1-MacA (Fig. 6B) and DL1 (Fig. 6D) cell walls show a thin uranium-bearing inner membrane, uranium-loaded periplasm and a very thin outer membrane. Dark-field TEM images (Fig. 6C, for strain DL1-MacA and E for strain DL1), that were obtained using the inner diffraction ring of SAED, show the bright feature of the cell wall, which confirms that the cell wall layers contribute to the diffuse diffraction rings in the SAED pattern. HRTEM images (Fig. 6F and 6G) show the amorphous features of the periplasmic uranium. In some local areas, several lattice fringes were observed in the uranium-bearing periplasm, most likely the result of electron beam damage. No uraninite nano-crystals were detected by TEM analysis. EDS analysis of the elemental composition of periplasmic space and cytoplasm yielded similar results for strains DL1 and DL1-MacA. A representative EDS pattern is shown on Fig. 7. The cytoplasm contained C, O and, to much smaller extent, P and U (Fig. 7A), while the periplasm was characterized by elevated concentrations of U and P (Fig. 7B), suggesting that periplasmic uranium was associated with phosphoryl and carbonyl ions. In previous studies, in which metal sequestration inside Gram-negative cells was studied, the metals accumulated in the periplasm were also found in association with phosphorus and carbon [39,41,42].

Our results demonstrate that uranium accumulated to a similar extent in the periplasm of U(VI) reduction-impaired mutants and wild type *G. sulfurreducens*. This implies that periplasmic uranium accumulation is unrelated to the capacity for uranium reduction and may instead reflect the ability of uranium to penetrate the outer membrane and react with substances in the periplasm that promote formation of precipitates. The valence of uranium in the periplasm of U(VI)-reducing *G. sulfurreducens *cells is difficult to determine unless it forms uraninite, and no uraninite nanominerals were detected by TEM. Our results contrast with recent findings of 1 – 5 nm uraninite nanoparticles in the periplasm of another U(VI)-reducing model organism *Shewanella oneidensis *[15]. A possible explanation for this difference could be the time at which uranium-reducing cell suspensions were sampled for TEM analysis (2 hours in our experiments vs 24 hours in experiments with *S. oneidensis*). Once uranium accumulates in the periplasm, it can be reduced by a network of periplasmic cytochromes, abundant in both *G. sulfurreducens *and *S. oneidensis*.

### Correlation between U(VI) and Fe(III)-reducing activity

**Figure 8:**
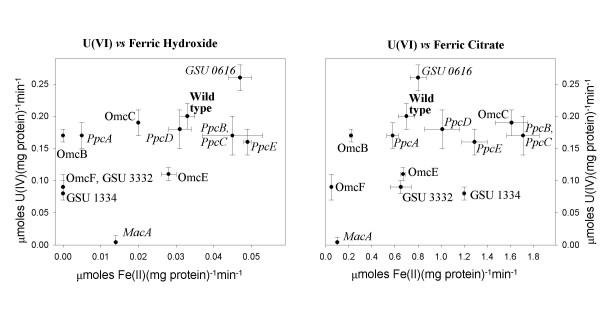
**Impact of cytochrome deletion on U(VI) and Fe(III) reduction**. Data are means of triplicates ± SD.

In order to gain insight into the relationship between the electron transport pathways leading to Fe(III) and U(VI), the ability of the various mutant cell suspensions to reduce both soluble and insoluble forms of iron was assessed. The impact of cytochrome mutations on U(VI) reduction did not necessarily correlate with their effect on reduction of Fe(III) hydroxide or soluble, chelated Fe(III) (Fig. 8). For example, a knock-out mutation of the *omcB *gene, which codes for an outer-membrane (OM) cytochrome required for Fe(III) reduction [25], had no impact on U(VI) reduction. On the other hand, the mutant DLMC8, in which the *omcE *gene was deleted, had low U(VI)-reducing, but wild type Fe(III)-reducing activity. Cell suspensions of mutants, DLMC5 and DLMC6, in which genes coding for putative OM cytochromes GSU1334 and GSU3332 were disrupted, were deficient in the reduction of U(VI) and Fe(III) hydroxide but not Fe(III) citrate.

There were also several cases, in which elimination of cytochromes had similar effects on U(VI)- and Fe(III)-reducing activity. For example, the OmcC-deficient strain could catalyze the reduction of all three electron acceptors at wild type or greater than wild type rates. Similarly, deletion of the putative periplasmic cytochrome GSU0616 increased the ability of cell suspensions to reduce all three acceptors. The MacA deficient strain was essentially incapable of reducing both U(VI) and Fe(III) citrate. However, it could still reduce Fe(III) oxides at 42% of the wild type rate.

Although elimination of some cytochromes impacted both U(VI) and Fe(III) reduction similarly, the general lack of correspondence between the impact of cytochrome knockouts on the reduction of the two acceptors, (Fig. 8) suggested that there are differences in the electron transfer pathways to these two metals.

This report constitutes the first characterization of the Fe(III)hydroxide reducing activity of periplasmic cytochromes. The impact of periplasmic cytochrome deletions on soluble and insoluble Fe(III) reduction was not always similar. Specifically, deletion of MacA had a greater impact on soluble Fe(III) reduction (86% decrease) than on Fe(III) hydroxide reduction (58% decrease). In contrast, the effect of deleting PpcA on the reduction of Fe(III) hydroxide was greater than its effect on soluble Fe(III) reduction. Elimination of PpcD increased the rate of soluble Fe(III) reduction, without significantly affecting the rate of insoluble Fe(III) reduction. There were also discrepancies between the rates of soluble and insoluble Fe(III) reduction by strains deficient in outer membrane cytochromes. These results indicate that the electron transport pathways to soluble and insoluble Fe(III) are likely to consist of a mixture of both common and unique elements.

### Interpretation of the impact of mutations on U(VI)- and Fe(III)-reducing activity

Given the lack of complementation studies for nearly half (5 out of 12, Table 1), of the cytochrome-deficient mutants described in this study, the observed differences in the U(VI)- and Fe(III)-reducing abilities of various cytochrome-deficient mutants should be regarded with caution. However, except for GSU3332 and GSU0616, all of the cytochromes for which complementation studies are not available are predicted to be transcribed monocistronically or at the 3' ends of operons (Table 1), where insertion of antibiotic resistance cassettes is less likely to cause polar effects. Another factor which may complicate interpretation of the results is the possibility of indirect effects on U(VI) reduction. For example, biochemical analysis revealed that deletion of the *omcF *gene altered the abundance of as many as six outer membrane *c*-type cytochrome proteins, including OmcB and OmcC [24]. Because deletion of OmcB negatively impacts Fe(III) reduction, the inability of the OmcF-deficient strain to reduce Fe(III) may be due to the lack of OmcB expression in this strain [25]. However, failure to express OmcB does not explain the reduction in the ability of the OmcF strain to reduce U(VI), since elimination of OmcB has little impact on U(VI) reduction (Fig. 4). Additional genetic and biochemical data will be needed to confirm the direct involvement of each of the cytochromes in U(VI) reduction.

## Conclusion

In conclusion, our results indicate that both periplasmic and outer membrane *c*-type cytochromes play a critical role in U(VI) reduction by *G. sulfurreducens*. In addition, we demonstrate that accumulation of amorphous uranium in the periplasm of U(VI)-reducing *G. sulfurreducens *cultures does not correlate with U(VI)-reducing activity. Our results provide new evidence for outer membrane U(VI) reduction in *G. sulfurreducens*, but do not rule out the possibility of periplasmic U(VI) reduction. Deletion of cytochromes rarely had a similar impact on both Fe(III) and U(VI) reduction, suggesting that there are differences in the routes of electron transfer to U(VI) and Fe(III). Further studies are required to clarify the pathways leading to U(VI) reduction in *G. sulfurreducens*.

## Methods

### Bacterial strains, plasmids, and culturing conditions

The bacterial strains that were used in this study are described in the Table 1. All strains were obtained from our laboratory collection. Construction and isolation of strains: DL1-MacA, DL3, DL5, DL6, DLLD2, DLLD3, DLLD4, DLMC8 and DLBKO1 has been previously described (references provided in Table 1.) Strains DLMC5, DLMC6, and DLMC7 were constructed as described below. *G. sulfurreducens *strains were routinely cultured anaerobically in either acetate:fumarate or acetate:Fe(III)-citrate medium as previously described [14,23].

### DNA manipulations and reagents

*G. sulfurreducens *genomic DNA was extracted using the MasterPure complete DNA & RNA purification kit (Epicentre Technologies, Madison, WI). Plasmid purification, PCR product purification, and gel extractions were performed with the following kits: the QIAprep Spin Miniprep Kit, the QIAGEN Plasmid Midi Kit, the QIAquick PCR Purification Kit, and the QIAquick Gel Extraction kit (QIAGEN Inc., Valencia, CA). Routine DNA manipulations were carried out according to the methods outlined by Sambrook *et al. *[43]. Restriction enzymes were purchased from New England Biolabs (Beverly, MA). Southern blot analyses for genotype confirmation were performed as previously described [23]. Taq DNA polymerase (Qiagen Inc.) was used for all PCR amplifications.

### Construction of cytochrome-deficient strains: DLMC5, DLMC6, DLMC7, and DLLD4

Linear fragments for the creation of the four cytochrome-deficient strains by homologous recombination were constructed by recombinant PCR [44] utilizing the primers listed in Table 3 as was previously described [25,29,45]. In strains DLMC5, DLMC6, and DLMC7, the majority of the cytochrome coding sequences were substituted with the kanamycin resistance cassette of pBBR1MCS-2 [46]. In strain DLLD4, the majority of the *ppcB *and *ppcC *genes and the intergenic region were replaced with by the chloramphenicol resistance cassette of pJRC2 [47]. In all four strains, the orientation of the antibiotic resistance cassette was the same as that of the disrupted genes. Electroporation, mutant isolation, and genotype confirmation were performed as described by Coppi *et al*. [23] and Lloyd *et al*. [27]. One each of the resulting mutants was chosen as the representative strain.

### Preparation of resting cell suspensions and measurement of U(VI) and Fe(III) reduction

The previously published protocol [21] for preparing resting cells and performing cell suspension experiments was modified as follows. *G. sulfurreducens *strains were cultured in anaerobic basal bicarbonate buffered (FW) medium [14] amended with 20 mM fumarate and 10 mM acetate instead of NB medium containing 40 mM fumarate and 15 mM acetate [23]. Late logarithmic phase cultures were harvested by centrifugation and washed twice in the following osmotically balanced wash buffer (g/L): NaHCO_3 _(2.5), NH_4_Cl (0.25), NaH_2_PO_4·_H_2_O (0.006), KCl (0.1), NaCl (1.75) instead of 30 mM bicarbonate. U(VI)- and Fe(III)-reducing activities were quantified using the following reaction buffer (g/l): NaHCO_3 _(2.5), NH_4_Cl (0.25), NaH_2_PO_4·_H_2_O (0.006), KCl (0.1). Cells were added to the reaction buffer to give a final protein concentration of 0.028 – 0.038 mg/ml for measuring Fe(III)-reducing activity and 0.007 – 0.017 mg/ml for measuring U(VI)-reducing activity instead of 0.25 mg/ml as previously described [21]. Concentrations of viable cells were determined using acridine orange staining and epifluorescent microscopy [40,48]. To produce the killed control (Fig. 2B), cell suspensions were autoclaved prior to addition to the reaction buffer.

The ability of cells to reduce Fe(III) was determined using either poorly crystalline ferric hydroxide (20 mM) or ferric citrate (20 mM) as the electron acceptor and 5 mM acetate as the electron donor. Fe(II) concentrations were determined for 0.1 ml samples taken at one hour intervals with the ferrozine assay as previously described [49].

The ability of cells to reduce U(VI) was determined using 1 mM uranyl acetate as the electron acceptor and 5 mM acetate as the electron donor as previously described [16,50]. U(VI) concentrations were quantified via Kinetic Phosphorescence Analysis (KPA) (Chemchec Corp., LaBrea, CA). Samples (0.5 ml) were taken at one hour intervals and diluted in 9 ml of anoxic 100 mM bicarbonate. The rate of enzymatic U(VI) reduction was calculated by subtracting the amount of U(VI) present in the diluted samples from the total amount of extractable uranium [U(IV) +U(V) + U(VI)] at each time point. To determine total extractable uranium, diluted samples were bubbled with air for 15 minutes in order to re-oxidize U(IV) and/or U(V) to U(VI).

### Transmission electron microscopy (TEM)

Cells were harvested from U(VI)-reducing cell suspensions after 2 hr of incubation in the presence of U(VI) by centrifugation at 6,000 × g for 10 min and washed once with the washing buffer described above. These preparations were fixed with 2% glutaraldehyde for 2 hr at 4°C, washed twice in 100 mM HEPES buffer, pH 7.0, enrobed in 2% (wt/vol) noble agar, and dehydrated in series of ethanol baths (25–100% (vol/vol)). Samples were infiltrated with 50% (vol/vol) LR White (Marivac, Ltd., Halifax, Canada) for 4 hours, 100% LR White overnight, and then embedded in 100% LR White for 1 h at 60°C for polymerization. Samples were thin-sectioned with a Reichert OM U4 Ultracut ultramicrotome. Sections were collected on copper grids (mesh size, 200 μM) covered with a carbon-coated Formvar film and examined with a transmission electron microscope (Phillips EM10) operated under standard conditions at 80 kV. Since no electron microscopy stains (such as osmium tetroxide, uranyl acetate, or lead citrate) were used, any contrast observed in the sections was due solely to the uranium bound by the cells.

SAED and HRTEM studies were carried out with a Philips CM 200 UT (Spherical aberration coefficient = 0.5 mm; Point to point resolution = 0.19 nm) equipped with NORAN Voyager X-ray energy-dispersive spectroscopy (EDS) at the Materials Science Center of the University of Wisconsin – Madison. The acceleration voltage was 200 kV. Bright-field (BF) TEM images were obtained by allowing only the direct beam to form images. Dark-field (DF) TEM images were obtained by allowing only the diffraction beam to form images using a small objective lens aperture. HRTEM images were obtained by using a large objective lens aperture and allowing both direct beam and diffraction beams to form images [51,52]

## Authors' contributions

ESS developed and carried out uranium and iron reduction assays for wild type and mutant strains of *G. sulfurreducens *and drafted the manuscript. MVC participated in designing experiments and critically reviewed the manuscript. MVC constructed strains DLMC5, DLMC6, and DLMC7 and LND constructed strain DLLD4. AAK carried out Transmission Electron Microscopy studies; SAS assisted in the preparation of cell suspensions and iron reduction assays. HK and HF carried out EDS, SAED, BF/DF TEM imaging, and HRTEM studies. LND, CL, JEB, and B-CK provided *G. sulfurreducens *mutants and participated in designing experiments. DRL conceived of the study, participated in its coordination and helped to draft the manuscript. All authors read and approved the final manuscript.

## Figures and Tables

**Table 1:** Strains used in the study.

Strain name	Genotype	Genbank accession # of deleted gene(s)	Predicted^b ^or confirmed^c ^Transcription unit(s) (5' to 3')	Source or reference
DL1 (ATCC 51573)	Wild type	-	-	[53]
DL5	*omcC*::*kan*	GI-39997825	1) *orf1orf2omcC*^c ^ 2) *omcC*^c^	[25] [54]
DL6^*a*^	*omcB::cam*	GI-39997831	1) *orf1orf2omcB*^c ^ 2) *omcB*^c^	[25] [54]
DLMC8^a^	*omcE::kan*	GI-39995725	1) *omcE*^*b*^	[35]
DLBK01^*a*^	*omcF::kan*	GI-39997527	1) *gsu2433*, *omcF*, *gsu2431*, *gsu2430*^*c*^	[24]
DLMC6	*gsu3332::kan*	GI-39998421	1) *gsu3332*, *gsu3331*, *gsu3330*^*b*^	This work
DLMC5	*gsu1334::kan*	GI-39996436	1) *gsu1336*, *gsu1334*^*b*^	This work
DLLD2^*a*^	*ppcE::kan*	GI-39996859	1) *gsu1758*, *gsu1759*, *ppcE*^*b*^	[28]
DLLD4^*a*^	*ppcBppcC::kan*	GI-39995472 GI-39995473	1) *ppcB, gsu0363*^*b *^ 2) *ppcC*^*b*^	This work (L. Didonato, personal communication)
DL3^*a*^	*ppcA::kan*	GI-39995719	1) *ppcA*^*b*^	[27]
DLLD3^*a*^	*ppcD::kan*	GI-39996127	1) *ppcD*^*b*^	[28] (C. Leang, personal communication)
DLMC7	*gsu0616::kan*	GI-39995723	1)*gsu0615, gsu0616, gsu0617*^*b*^	This work
DL1-MacA	*macA::kan*	GI-39995574	1) *macA*^*c*^	[26]

^a^Strains for which impairment in growth via Fe(III) reduction was complementation complemented by expression of the relevant cytochrome gene *in trans*. In the case of strain DLMC8, impairment in growth on Fe(III) oxide was complemented by expression of OmcE *in trans*. In all other cases Fe(III) citrate phenotypes were complemented. The Fe(III) citrate reduction phenotype of the double mutant, DLLD4 was complemented by expressing *ppcB in trans*.

^b^Transcription units were predicted with the FGENESB software package (Softberry Inc., Mt. Kisco, NY) as previously described [55]. The numbers 1) and 2) indicate distinct, predicted transcripts.

^c^Transcription units have been experimentally confirmed by Northern blot. The numbers 1) and 2) refer to distinct, experimentally confirmed transcripts.

**Table 2:** Localization of cytochromes

Protein IDs	Cellular Localization (p/c*)	Sources/References
OmcC	OM ** (**p**)	PSORT [56]
OmcB	OM (**c**)	[36]
OmcE	OM (**c**)	[35]
OmcF	OM (**c**)	[24]
GSU3332	OM (**p**)	PSORT [56]
GSU1334	OM (**p**)	PSORT [56]
PpcA	P **(**c**)	[31]
PpcB	P (**p**)	Proteome analyst [33]
PpcC	P (**p**)	Proteome analyst [33]
PpcD	P (**p**)	Proteome analyst [33]
PpcE	P (**p**)	Proteome analyst [33]
GSU0616	P (**p**)	Proteome analyst [33]
MacA	P (**p**)	Proteome analyst [33]

*Subcellular localization of cytochromes was either (**c**) confirmed by biochemical analyses or immunoelectron microscopy or (**p**) predicted using one of two servers, PSORT [57] or Proteome analyst v2.5 [32].

** OM – outer membrane; P – periplasmic.

**Table 3:** Primers used in the study

Target cytochrome gene(s)	Primer name	Primer sequence (5' to 3')
*ppcB, ppcC*	ppc.4.1B	GTCCGCTCTGCCTTC AG
	ppc.4.2	CATGGTCACACTCCTTTG C
	ppc.4.3cam	GAGATTCATTCCAGCAACCGCGACGGAAGATCACTTCGC
	ppc4.4cam	GTCGGAATTACTTCTTGTGGCAGGGCACCAATAACT GC
	ppc4.5cam	GCAAGGGGTGTCACGAGG
	ppc4.6	GCAAGGGGTGTCACGAGG
GSU0616	pr1027-1	CTGAGCTACGAGCAGAATGAC
	pr1027-2	CGACGAGATAGCCAGCATATC
	pr1027-3	CGGACCATCTGGTTCTCAAGAATGTCAGCTACTGGGCTATC
	pr1027-4	GGCAACGGTCTCATGGCAGGAATCGAAATCTCGTGATGG
	pr1027-5	CCTGCCATGAGACCGTTGCC
	pr1027-6	GCTCATGGCCTGTTCTTCTCC
GSU1334	pr2262-1	TCAGCGCCTTCGTGGATTCG
	pr2262-2	GTGGACTGAAAGCTGGGAACG
	pr2262-3	CGACACCGTTCCCAGCTTTCAGCAAGCGAACCGGAATTGC
	pr2262-4	CCTTGAACGGGACGATGATGGGATGAATGTCAGCTACTGG
	pr2262-5	ACCACTATCCGGAGCTGCGAG
	pr2262-6	CCAGGAGATGACCGTTGCTTCG
GSU3332	pr5560-1	TCA AGG AGT TGG GCC GAA TCC
	pr5560-2	CGA CAG CAT GGC CAC GAA TG
	pr5560-3	CACACTTCTGGCATTCGTGGATGAATGTCAGCTACTGG
	pr5560-4	CGTCCACCTTGCGGATGTCGAATCGAAATCTCGTGATGG
	pr5560-5	CGACATCCGCAAGGTGGACG
	pr5560-6	CGTCCAGCAGGATGCGTGAG
